# *In Vitro* Evaluation of Curcumin-Encapsulated Chitosan Nanoparticles against Feline Infectious Peritonitis Virus and Pharmacokinetics Study in Cats

**DOI:** 10.1155/2020/3012198

**Published:** 2020-05-31

**Authors:** Shing Wei Ng, Gayathri Thevi Selvarajah, Mohd Zobir Hussein, Swee Keong Yeap, Abdul Rahman Omar

**Affiliations:** ^1^Institute of Bioscience, Universiti Putra Malaysia, 43400 UPM Serdang, Selangor, Malaysia; ^2^Department of Veterinary Clinical Studies, Faculty of Veterinary Medicine, Universiti Putra Malaysia, 43400 UPM Serdang, Selangor, Malaysia; ^3^Laboratory of Materials Synthesis and Characterization, Institute of Advanced Technology, Universiti Putra Malaysia, 43400 UPM Serdang, Selangor, Malaysia; ^4^China-ASEAN Marine Science School, Xiamen University Malaysia, Sepang, Selangor, Malaysia; ^5^Department of Pathology and Microbiology, Faculty of Veterinary Medicine, Universiti Putra Malaysia, 43400 UPM Serdang, Selangor, Malaysia

## Abstract

Feline infectious peritonitis (FIP) is an important feline viral disease, causing an overridden inflammatory response that results in a high mortality rate, primarily in young cats. Curcumin is notable for its biological activities against various viral diseases; however, its poor bioavailability has hindered its potential in therapeutic application. In this study, curcumin was encapsulated in chitosan nanoparticles to improve its bioavailability. Curcumin-encapsulated chitosan (Cur-CS) nanoparticles were synthesised based on the ionic gelation technique and were spherical and cuboidal in shape, with an average particle size of 330 nm and +42 mV in zeta potential. The nanoparticles exerted lower toxicity in Crandell-Rees feline kidney (CrFK) cells and enhanced antiviral activities with a selective index (SI) value three times higher than that of curcumin. Feline-specific bead-based multiplex immunoassay and qPCR were used to examine their modulatory effects on proinflammatory cytokines, including tumour necrosis factor (TNF)*α*, interleukin- (IL-) 6, and IL-1*β*. There were significant decrements in IL-1*β*, IL-6, and TNF*α* expression in both curcumin and Cur-CS nanoparticles. Based on the multiplex immunoassay, curcumin and the Cur-CS nanoparticles could lower the immune-related proteins in FIP virus (FIPV) infection. The single- and multiple-dose pharmacokinetics profiles of curcumin and the Cur-CS nanoparticles were determined by high-performance liquid chromatography (HPLC). Oral delivery of the Cur-CS nanoparticles to cats showed enhanced bioavailability with a maximum plasma concentration (*C*_max_) value of 621.5 ng/mL. Incorporating chitosan nanoparticles to deliver curcumin improved the oral bioavailability and antiviral effects of curcumin against FIPV infection. This study provides evidence for the potential of Cur-CS nanoparticles as a supplementary treatment of FIP.

## 1. Introduction

Feline infectious peritonitis (FIP) was first recognised as an important disorder in cats in 1963 [1]. FIP virus (FIPV), which is classified as a feline coronavirus (FCoV) of the family *Coronaviridae*, is known to be the causative agent of this deadly disease. FCoVs are further differentiated into two distinct serotypes based on virus-neutralising antibodies [2]. Type I FCoVs are the most encountered virus in the field and are the likely cause of clinical FIP worldwide, while type II FCoVs, which are closely related to canine coronaviruses, constitute more than 25% prevalence in Asia and are studied mostly *in vitro* [2–4]. FIP is a fatal immune-mediated viral disease, often overwhelming cytokine production, and the intense granulomatous inflammatory response causes damage to the host, where pleural and abdominal effusion are present clinically in some cases [5]. Its immune evasion mechanisms mean FIPV can elude clearance by the host's immune response, thus aggravating the progression of disease [6].

For over half a century, numerous studies have investigated the therapeutic effects of various antiviral agents and immunomodulatory drugs against FIPV infection, but most were to no avail [7–9]. The viral inhibitory effect of chloroquine, an antimalarial drug, has been investigated *in vitro* and *in vivo*; it could inhibit FIPV replication and reduce inflammatory cytokine levels, yet it caused severe adverse effects when used at higher dosages [10]. Vaccination against FIPV had promoted its infectivity to cats rather than providing protection, particularly because the existence of non-neutralising antibodies had facilitated FIPV infection [11, 12]. DNA vaccines were incapable of inducing cell-mediated immunity against FIPV challenge, and co-administration of interleukin- (IL-) 12 with the vaccines further caused adverse effects to the cats [13]. Nevertheless, a recent study showed itraconazole, a common antifungal drug, was effective for inhibiting infection by type I FCoV [14]. Other promising anti-FIPV drugs, e.g., GC376 3C-like protease inhibitor and nucleoside analogue GS-441524, were reported to have significant antiviral effects in naturally occurring FIP in cats [15, 16].

The antiviral properties of curcumin against different families of viruses such as human immunodeficiency virus (HIV), influenza virus H1N1, Ebola virus, and severe acute respiratory syndrome coronavirus (SARS-CoV) have been demonstrated [17–20]. Curcumin is a phenolic compound derived from the rhizome of a turmeric known as *Curcuma longa*. Curcumin has a wide spectrum of pharmacological activities, including antiangiogenic, antibacterial, antioxidant, and anti-inflammatory activities [21–24]. In addition, many studies on curcumin-associated toxicity in human and animal models have reported that curcumin is safe for consumption even at high doses without apparent adverse effects [25, 26].

Despite the efficacy and safe use of curcumin in various diseases being well documented, the poor bioavailability of this agent is a major concern, particularly for its therapeutic application. Studies to date have revealed low plasma and tissue levels of curcumin due to poor absorption, rapid metabolism, and rapid systemic elimination, thus severely disrupting its efficacy as a treatment choice [27, 28]. Countless efforts have been made to improve the bioavailability of curcumin by incorporating different methods and formulations such as micelles, liposomes, hydrogels, inorganic nanoparticles, and polymeric nanoparticles with the aim of enhancing its permeability and resistance to the metabolic processes [28–32].

Chitosan is a cationic linear polysaccharide composed of linked glucosamine units with some proportion of N-acetylglucosamine units, which are derived from chitin. Chitosan has gained considerable attention primarily due to its biodegradable, biocompatible, low-toxicity and mucoadhesive properties [33, 34]. Chitosan-based nanocarriers including nanoparticles, nanofibers, nanogels, and nanocomposites favour diverse features and characteristics for effective encapsulation of bioactive compounds and nutraceuticals with enhanced bioavailability and stability [35]. Chitosan-based nanoparticles have been employed as a delivery system for insulin, verapamil hydrochloride, small interfering RNA-based drugs, and other hydrophobic drugs by providing various routes of administration, i.e., via ocular, intranasal, and oral routes, and even as an antigen delivery system, they also enhance drug permeability and resistance to the metabolic processes [36–39].

The present study reports the antiviral and anti-inflammatory activities of curcumin-encapsulated chitosan (Cur-CS) nanoparticles against FIPV infection in Crandell-Rees feline kidney (CrFK) cells in addition to a pharmacokinetics study of the related compounds in cats.

## 2. Materials and Methods

### 2.1. Materials and Reagents

Curcumin powder, low-molecular weight chitosan (75–85% deacetylated), Tween 80, sodium tripolyphosphate (TPP), sucrose, and sodium hydroxide (NaOH) were purchased from Sigma-Aldrich (St Louis, MO, USA). Acetic acid, ethyl acetate, and methanol were purchased from AMRESCO (Radnor, PA, USA). Absolute ethanol was purchased from Scharlau (Barcelona, Spain). All reagents utilised in this study were of analytical grade, except methanol, which was high-performance liquid chromatography (HPLC) grade. CrFK cells and FIPV strain WSU 79-1146 were purchased from ATCC (Manassas, VA, USA). Minimum essential medium (MEM) containing Earle's salts and L-glutamine, foetal bovine serum, nonessential amino acid (NEAA), penicillin (100 U/mL), streptomycin (100 *μ*g/mL), phosphate-buffered saline (PBS), TryPLE™, trypan blue, dimethyl sulfoxide (DMSO), and 3-(4,5-dimethyl-2-thiazolyl)-2,5-diphenyl-2H-tetrazolium bromide (MTT) were purchased from Thermo Fisher Scientific (Waltham, MA, USA).

### 2.2. Preparation of Cur-CS Nanoparticles

Chitosan nanoparticles were prepared via ionotropic gelation, where chitosan was dissolved in 2% *v*/*v* acetic acid to yield a 0.1% *w*/*v* chitosan solution, which was adjusted to pH 3 with 2 M NaOH. Then, TPP (1 mg/mL) prepared in double-distilled water was added into the chitosan solution dropwise in the ratio of 1 to 5 (TPP to chitosan) under constant magnetic stirring conditions at room temperature for 2 h [40, 41]. The nanoparticle suspension was then centrifuged at 12,000 g for 30 min. The supernatant was discarded, and the nanoparticles were resuspended in Millipore water.

Curcumin was dissolved in ethanol to yield a final concentration of 5 mg/mL. Different concentrations of curcumin were added to a fixed concentration of chitosan nanoparticles in ratios of 0.5 : 1, 0.75 : 1, and 1 : 1 where the curcumin solution was incubated with a 500 mg chitosan nanoparticle suspension in 40 mL Millipore water at pH 3, adjusted by adding 2 M NaOH. The mixture was stirred at 1,000 rpm for 12 h at 37°C. The unbound curcumin was removed by centrifugation at 12,000 g at 4°C for 30 min, and the pellet was washed three times with 10% aqueous ethanol. The nanoparticles were then freeze-dried and stored at 4°C.

### 2.3. Encapsulation Efficiency and Loading Capacity

The loading capacity and encapsulation efficiency of the 0.5 : 1, 0.75 : 1, and 1 : 1 curcumin to chitosan nanoparticle ratios were measured using an Ultrospec 3000 pro Ultraviolet-Visible (UV-Vis) spectrophotometer (Pharmacia Biotech, Peapack, NJ, USA). A curcumin calibration curve (*R*^2^ = 0.992) at a 427 nm wavelength was constructed. Encapsulation efficiency was calculated using the formula as below during the Cur-CS nanoparticle preparation process. As for the drug loading capacity, curcumin was extracted from a weighed amount of Cur-CS nanoparticles as previously described [40]. The concentration of extracted curcumin solution was then quantified using the UV-Vis spectrophotometer and calculated using the existing curcumin calibration curve. The drug encapsulation efficiency and drug loading capacity of the nanoparticles were calculated as follows:

(1) $$\begin{array}{c} \text{Encapsulation efficiency}=\frac{A-B}{A}\times 100\%,\\ \text{Drug loading capacity}=\frac{C}{D}\times 100\%, \end{array}$$

where *A* is the total amount of curcumin used, *B* is the amount of free curcumin available in the supernatant, *C* is the amount of curcumin released from the nanoparticles, and *D* is the weight of the nanoparticles.

### 2.4. Characterisation of Cur-CS Nanoparticles

The particle size and zeta potential of the nanoparticles were measured based on dynamic light scattering (DLS) using a Zetasizer® Nano ZS (Malvern Instruments, Worcestershire, UK). The morphology of the nanoparticles was examined using a transmission electron microscope (TEM, Hitachi H-7100, Tokyo, Japan). The nanoparticle solution was sonicated for 2 min to prevent particle aggregation, then one drop of the solution was placed on carbon film-coated copper grids and air-dried at room temperature. The excess fluid was removed with filter paper. The samples were then viewed under TEM, and the images were captured.

### 2.5. Release Study

Sampling vials each containing 5 mg Cur-CS nanoparticles dissolved in 5 mL PBS with 1% (*v*/*v*) Tween 80 (pH 7.4) were prepared. The tubes were incubated in a water bath shaker at 200 rpm and 37°C. At 0.5, 1, 2, 4, 8, 12, and 24 h intervals, one set of tubes was removed and then centrifuged at 1300 g for 5 min (Allegra™ X22R Centrifuge, Beckman Coulter, La Brea, CA, USA). The amount of curcumin released in the supernatant was quantified spectrophotometrically at the 427 nm wavelength. The percentage of drug released was calculated as follows:

(2) $$\begin{array}{c} \text{Percentage released }\left(\%\right)=\frac{E}{F}\times 100\%, \end{array}$$

where *E* is the amount of curcumin released and *F* is the total curcumin in the Cur-CS nanoparticles.

### 2.6. Stability Testing

The average particle size and zeta potential of Cur-CS nanoparticles placed at room temperature (ca. 25°C) and 4°C were measured at day 0 (before storage), day 15, and day 30. The nanoparticles were dissolved in PBS before being measured using the Zetasizer® Nano ZS. The average mean nanoparticle size was used as an indicator to evaluate their physical stability.

### 2.7. Cellular Uptake Study

The cellular uptake of curcumin and the Cur-CS nanoparticles by CrFK cells was examined using the fluorescence method. Briefly, CrFK cells at a density of 1.5 × 10^5^ were grown in MEM supplemented with 10% foetal bovine serum, 1% NEAA, 1% penicillin (100 U/mL), and 1% streptomycin (100 *μ*g/mL) in coverslip-mounted 6-well plates. The plates were incubated at 37°C in an incubator with 5% CO_2_ for 24 h. Then, 20 *μ*M curcumin and Cur-CS nanoparticles were added to the respective wells and incubated for 0.5, 1, 3, 6, and 12 h. At these intervals, the cells were rinsed three times with PBS. The coverslips with the attached cells were removed, fixed with 2% paraformaldehyde, and dried overnight in the absence of light. The dried coverslips were mounted on a glass slide, and images were captured by an Olympus IX81 inverted fluorescence microscope (Olympus, Japan) under a 535–600 nm filter.

### 2.8. Cytotoxicity Assay

CrFK cells at a density of 2 × 10^4^ were incubated in 96-well culture plates for 24 h at 37°C. Then, the cells were washed with PBS. Twofold serial dilutions of curcumin, Cur-CS nanoparticles, and chitosan nanoparticles were added to the semiconfluent cells and incubated for 24, 48, and 72 h. A colorimetric MTT assay was performed. Briefly, 20 *μ*L 5 mg/mL MTT solution was added to each well and incubated for 2 h at 37°C. Then, 170 *μ*L solution was removed from each well; subsequently, 100 *μ*L DMSO was added to dissolve the formazan crystals. The plate was then incubated for 1 h before optical density (OD) measurement at 570 nm using a plate reader (BioTek EL800, BioTek, Winooski, VT, USA).

The relative cell viability (%) of CrFK cells after treatment was calculated using the formula (OD_570 (treated cells)_/OD_570 (control cells)_) × 100. The median cytotoxic concentration (CC_50_) is the concentration that reduces the absorbance of treated cells to 50% with respect to the cell controls. The CC_50_ of the compounds was calculated by regression analysis in SPSS software version 22.0 (IBM, Chicago, IL, USA).

### 2.9. Cytopathic Effect Inhibition Assay

The cytopathic effect (CPE) inhibition assay with slight modifications was used as previously described [42]. Time of addition tests were applied, where the CrFK cells were treated with different exposures (pre-, co-, and posttreatment) of the indicated concentrations of curcumin and Cur-CS nanoparticles. In pretreatment, the compounds were included in the cell culture medium for 8 h and were removed prior to virus infection. In cotreatment, the compounds were mixed with virus in the medium, added simultaneously to the cells, and left on the cells throughout. For posttreatment, the compounds were added to the cells 2 h postinfection (hpi) and remained throughout the time of infection. Briefly, CrFK cells were incubated in 96-well plates and incubated with predetermined concentrations of curcumin and Cur-CS nanoparticles under different types of exposure with 100 tissue culture infective dose 50 (TCID_50_) FIPV. After incubation at 37°C for 48 and 72 h, the cell viability was determined by MTT.

The percentage of protection was calculated based on the OD using the following formula:

(3) $$\begin{array}{c} \text{Percentage of protection }\left(\%\right)=\frac{{\text{OD}}_{570\;\left(\text{T}\right)}-{\text{OD}}_{570\;\left(\text{V}\right)}}{{\text{OD}}_{570\;\left(\text{C}\right)}-{\text{OD}}_{570\;\left(\text{V}\right)}}\times 100, \end{array}$$

where T is the treated sample, V is the virus-infected control, and C is the negative control.

The median effective concentration (EC_50_) is the concentration of compound required to achieve 50% protection against virus-induced CPE. The selective index (SI) was calculated for curcumin and Cur-CS nanoparticles against FIPV by dividing the appropriate CC_50_ value by the corresponding EC_50_.

### 2.10. Antiviral Assay in Cotreatment

CrFK cells at a density of 1 × 10^5^ were cultured in medium in 24-well plates for 24 h at 37°C. Then, the cells were incubated with 5, 10, and 20 *μ*M of curcumin and Cur-CS nanoparticles and 100 TCID_50_ FIPV in a coinoculation treatment, and the plates were incubated for 48 and 72 h. The culture supernatant was centrifuged at 700 g for 10 min, and the virus-containing supernatant was collected and used for virus quantification.

For the cytokine measurement study, the cells were incubated with 20 *μ*M curcumin and Cur-CS nanoparticles and 100 TCID_50_ virus by coinoculation treatment and incubated for 24 and 48 h. The culture supernatant was centrifuged and used for the bead-based multiplex immunoassay, while the attached cells were trypsinised and collected for the cellular proinflammatory cytokine mRNA expression study.

### 2.11. RNA Extraction

Viral RNA was extracted from the collected supernatant using a Viral Nucleic Acid Extraction Kit II (Geneaid, New Taipei City, Taiwan) following the protocol provided by the manufacturer. Cellular total RNA was extracted from the CrFK cell pellet using the RNeasy® Mini Kit, which includes DNase treatment (QIAGEN, Germantown, MD, USA). The concentration and purity of the extracted RNA were quantified using the Eppendorf BioSpectrometer® kinetic (Eppendorf, Hamburg, Germany). The extracted RNA was stored at -80°C until further use.

### 2.12. Complementary DNA Preparation and Viral mRNA Quantification

Complementary DNA (cDNA) was prepared, and viral mRNA was quantified using quantitative PCR (qPCR) employing previously described methods [43]. The qPCR results were quantified using the absolute quantification approach, where a standard curve of a serial dilution of viral cDNA template was plotted before the quantification.

### 2.13. Proinflammatory Cytokine mRNA Expression Study

The expression of three cytokine genes, namely, IL-1*β*, IL-6, and tumour necrosis factor (TNF)*α*, and a reference gene (glyceraldehyde-3-phosphate dehydrogenase, GAPDH) was determined by reverse transcriptase qPCR. The sequences of primers used have been reported previously and are listed in Table 1 [10, 44]. The reaction mix was prepared, where 10 *μ*L 2x SensiFAST™ SYBR® No-ROX One-Step Mix, 0.8 *μ*L 10 *μ*M forward primer, 0.8 *μ*L 10 *μ*M reverse primer, 0.2 *μ*L reverse transcriptase, 0.4 *μ*L RiboSafe RNase inhibitor, 5.8 *μ*L DNase/RNase-free water, and 2 *μ*L RNA sample (100 ng) made a total volume of 20 *μ*L (SensiFAST SYBR No-ROX One-Step kit, Bioline, London, UK). Then, the reaction mixture was run in a CFX96 Touch™ Real-Time PCR Detection System (Bio-Rad, Hercules, CA, USA) with the following cycling conditions: one cycle at 45°C of cDNA synthesis for 10 min, one cycle at 95°C of denaturation for 5 min, and 40 cycles of denaturation at 95°C for 15 s, annealing at 60°C for 15 s, and extension at 72°C for 15 s. The relative IL-1*β*, IL-6, and TNF*α* mRNA levels were calculated by the comparative threshold cycle (Ct) method using GAPDH as the reference gene.

### 2.14. Feline-Specific Magnetic Bead-Based Multiplex Immunoassay

Nineteen immune-related proteins including cytokines, chemokines, growth factors, and other immunologically active proteins were measured using a commercially available feline-specific magnetic bead-based multiplex immunoassay. The Feline Cytokine/Chemokine Magnetic Bead Panel Premixed (FCYTOMAG-20K-PMX; EMD Millipore, Billerica, MA, USA) was purchased and used according to the manufacturer's recommendation. Quality control samples, standards, and sets of culture sample were run on plates in duplicate. Prior to measurement, the culture samples were concentrated using Concentrator 5301 (Eppendorf, Hamburg, Germany). The plates were evaluated using MAGPIX® (Luminex, Austin, TX, USA); subsequently, the results were analysed using MILLIPLEX Analyst v.5.1 (EMD Millipore, Billerica, MA, USA).

### 2.15. Animal Study: Ethics and Animal Preparation

Animal ethics approval was obtained from the Institutional Animal Care and Use Committee (IACUC), Universiti Putra Malaysia (UPM/IACUC/AUP-R004/2015). A total of 14 young cats that fulfilled the inclusion criteria were selected. The cats (1) were clinically healthy; (2) aged 3–12 months; (3) had good haematology, serum biochemistry, and urine analysis profiles; and (4) had tested seronegative for FCoV, feline leukaemia virus (FeLV), and feline immunodeficiency virus (FIV) with commercially available kits (FIP antibody test kit, Biogal Galed Laboratories, Kibbutz Galed, Israel; SNAP FIV/FeLV combo test kit, IDEXX, Westbrook, ME, USA). All the cats were domestic short hair, aged 4–7 months, and there were nine males and five females.

### 2.16. Pharmacokinetics Study

Single-dosing (s.i.d.) and two-dosing (b.i.d.) oral administration of curcumin and Cur-CS nanoparticle pharmacokinetics study spanning 24 h was performed in the cats, which weighed 1.5–3.5 kg. The cats were randomly divided into four groups, and each group contained four cats. For Group A, curcumin (250 mg/kg) was orally administered s.i.d. For Group B, Cur-CS nanoparticles (250 mg/kg) were orally administered s.i.d. For Group C, curcumin (250 mg/kg) was given orally to cats b.i.d. 12 h apart. For Group D, Cur-CS nanoparticles (250 mg/kg) was administered orally b.i.d. 12 h apart. Prior to and 0.5, 1, 2, 3, 4, 8, 12, and 24 h after the treatments, 1 mL blood was harvested via jugular, cephalic, or saphenous venipuncture into lithium heparinized tubes (BD, Franklin Lakes, NJ, USA). The plasma was immediately centrifuged at 500 g for 5 min at 4°C and stored at -80°C until use. The cats then underwent a 2-week washout and rest period prior to testing on the following treatment and series of blood collection. All the cats were neutered at the end of the study and adopted by the public.

### 2.17. High-Performance Liquid Chromatography

Curcumin was extracted from plasma using the polar solvent extraction method. Ethyl acetate (500 *μ*L) was added to 200 *μ*L plasma and vortexed for 10 min. The samples were centrifuged at 4000 g at 10°C for 15 min, and the upper ethyl acetate layer was transferred to a new 1.5 mL tube. The extraction was repeated twice and performed under dim light conditions to prevent the degradation of curcumin. The combined ethyl acetate layers were then dried under vacuum, and the residue was dissolved in 100 *μ*L methanol. An aliquot of a 20 *μ*L sample was analysed for quantifying the curcumin in the plasma by HPLC.

The curcumin was quantified using a Series 1200 Quaternary HPLC system (Agilent Technologies, Santa Clara, CA, USA) with an UV-Vis detector equipped with an Eclipse Plus C18 column (Agilent Technologies). The HPLC system was first optimised as previously described and further adjusted to suitable settings [45]. The mobile phase was a mixture of 2% acetic acid at pH 3 and methanol at a proportion of 30 : 70, *v*/*v*, respectively. The flow rate of the mobile phase was maintained at 1 mL/min with an injection volume of 20 *μ*L sample, where the column temperature was maintained at 35°C. The detector signal of the HPLC system for curcumin detection was adjusted to 425 nm. A 6-point calibration curve was prepared by serial dilution of 1 mg/mL curcumin stock solution in methanol. Curcumin concentrations (ng/mL) in the plasma were calculated based on the calibration curve. Time to reach maximum plasma concentration (*T*_max_) and maximum plasma concentration (*C*_max_) values were obtained directly from the measured data. The area under the curve from 0 to *t* (AUC_0-*t*_) was calculated using the linear-log trapezoidal method.

### 2.18. Statistical Analysis

The experimental data were reported as the mean ± standard deviation (SD). Paired sample *t*-test and analysis of variance (ANOVA) post hoc Duncan multiple test were used for data analysis. All statistical analyses were performed using SPSS software version 22.0 (Chicago, IL, USA). Sample values between the different treatments and groups with *p* < 0.05 were to be statistically significantly different.

## 3. Results and Discussion

### 3.1. Preparation and Characterisation of Nanoparticles

**Figure 1:**
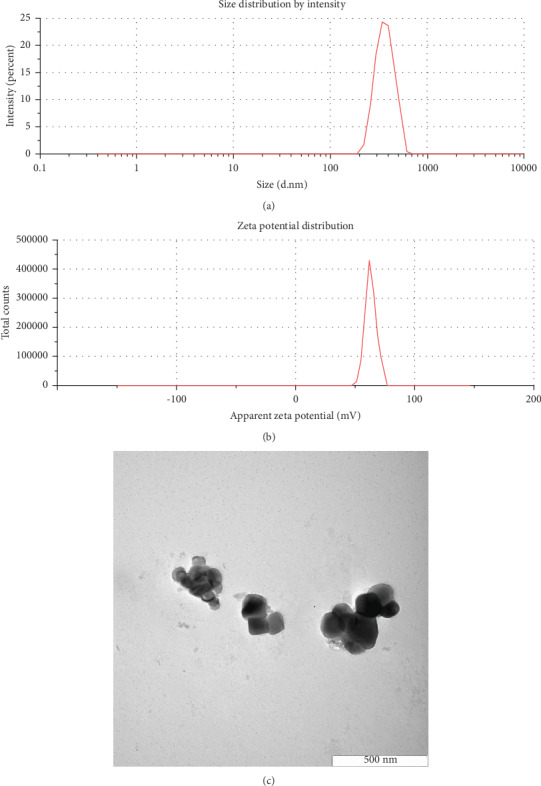
(a) Particle size distribution of Cur-CS nanoparticles, (b) zeta potential distribution of Cur-CS nanoparticles, and (c) TEM image of Cur-CS nanoparticles.

The preparation of chitosan nanoparticles via the ionic gelation technique is achieved by the noncovalent interaction between the positively charged chitosan and negatively charged polyanions, particularly TPP as the crosslinking agent, under acidic conditions. This formulation was first reported in 1997 and has been labelled as a promising delivery system for proteins, oligonucleotides, and bioactive molecules (hydrophobic, hydrophilic, or macromolecule) [39, 41]. The molecular weight of chitosan, degree of deacetylation, chitosan : TPP weight ratio, and pH greatly affect the physicochemical and biological properties of the synthesised chitosan nanoparticles [40, 46, 47]. Preliminary studies had determined that the ratio of chitosan to TPP at 5 to 1 produced smaller sizes of chitosan nanoparticles. DLS showed that chitosan nanoparticles produced with low-molecular weight chitosan had a mean particle size of 239 nm, with a zeta potential of +53.3 ± 2.6 mV. The Cur-CS nanoparticles were prepared by incubating chitosan nanoparticles with curcumin solution at different ratios. The Cur-CS nanoparticles synthesised with 0.75 : 1 chitosan solution : curcumin had a mean particle size of 332.4 nm, slightly larger than that of the chitosan nanoparticles (Figure 1(a)). Conversely, the nanoparticles had a lower zeta potential value, i.e., +42.1 ± 3.0 mV, compared to the chitosan nanoparticles (Figure 1(b)). The TEM imaging showed that the Cur-CS nanoparticles were spherical and cuboidal in shape and ranged in size from 250 nm to 450 nm (Figure 1(c)) and were in accordance with the mean particle size measured with DLS. Loading curcumin onto chitosan nanoparticles increasing the particle size is in agreement with previous studies; despite the lower surface charge compared to chitosan nanoparticles, +30 mV is sufficient for maintaining particle stability in a nanosuspension [40, 48, 49].

### 3.2. Curcumin Loading and Encapsulation Efficacy

In the present study, curcumin encapsulation efficiency and loading capacity were significantly influenced by the chitosan : curcumin concentration ratio used in the formation of the Cur-CS nanoparticles (Table 2). The encapsulation efficiency and loading capacity of 1 : 1 chitosan : curcumin was significantly lower when compared to ratios of 1 : 0.75 and 1 : 0.5. However, both parameters were not affected effectively when the Cur-CS nanoparticles were prepared with 1 : 0.75 and 1 : 0.5 chitosan : curcumin; there was also no significant difference in the measured mean particle size. Recent studies have demonstrated that curcumin-loaded chitosan nanoparticles produced by the ionic gelation technique exhibited particle sizes within the range of 100 to 500 nm and high encapsulation efficiency (>50%); however, pH sensitivity and high polydispersity are considered as the drawbacks of this method [31, 35]. The volume of TPP added during the process to strengthen the covalent bonds between chitosan nanoparticles and curcumin could affect the loading capacity, but several studies have pointed out that TPP has no substantial effect on the loading capacity; rather, it is due to other underlying causes [48, 50–52]. Cur-CS nanoparticles prepared at 1 : 0.75 chitosan : curcumin ratio with encapsulation efficiency of 77.2% and loading capacity of 46.7% were evaluated in subsequent experiments.

### 3.3. Curcumin Release Profile

**Figure 2:**
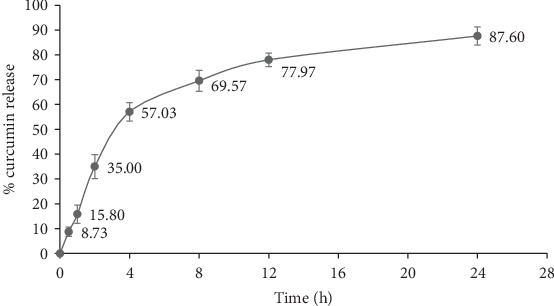
Curcumin release profile of Cur-CS nanoparticles within 24 h.

A 24 h curcumin release profile of the nanoparticles was studied (Figure 2). More than 50% of curcumin was released from the nanoparticles in the initial 4 h. The release rate of curcumin was gradually slower after 4 h, and a total of 87.6% curcumin was released from the nanoparticles within 24 h. A slower release rate in Cur-CS nanoparticles was observed as compared to a previous study, where a total of 80% curcumin was released from the particles over 6 h [51]. An initial rise in curcumin release within the first 4 h might have been due to the release of surface- and near surface-associated curcumin on the nanoparticles. Over time, curcumin began to release from the inner matrix of the nanoparticles once the surface-associated curcumin became depleted. Considering the covalent drug-polymer attachment, the time required for bond cleavage delays drug immediate action and prevents short-term drug release; thus, the deeper-residing curcumin requires a longer time for release to the surface [51, 53]. The degree of release from nanoparticles is associated with the composition of the formulation, drug molecular structure and its molecular weight, and the relative affinities of the drug and polymer and the aqueous phase [54, 55].

### 3.4. Stability Study

Freeze-drying is a common preservation method for nanoparticles to improve their physical and chemical instability and thus prolong the storage period [56]. To study the storage stability of the Cur-CS nanoparticles at 4°C and room temperature (ca. 25°C), the mean particle size and zeta potential were measured at days 0, 15, and 30, and no significant difference was observed in the mean particle size and zeta potential of Cur-CS nanoparticles stored at 4°C (Table 3). Cur-CS nanoparticles stored at room temperature yielded similar findings, despite significantly larger mean particle sizes noted after 15 and 30 days of storage. As the long-term physical stability of chitosan nanoparticles crosslinked with TPP is greatly affected by the ionic strength, chitosan concentration, and the TPP : chitosan ratio employed in the particle preparation, the data show Cur-CS nanoparticles can be stored at 4°C for 1 month without presenting any physical instability [57]. Storage temperature seems to have a vital impact on the long-term stability of biodegradable nanoparticles [56].

### 3.5. Cellular Uptake of Cur-CS Nanoparticles

**Figure 3:**
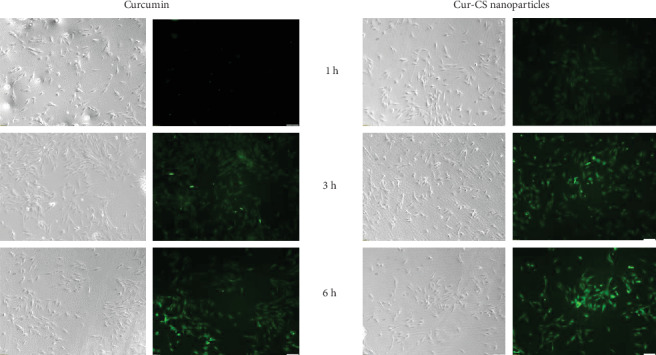
Phase contrast and fluorescence images of cellular uptake of curcumin and Cur-CS nanoparticles at different time intervals.

The autofluorescence property of curcumin eased the investigation on the cellular uptake of curcumin and the Cur-CS nanoparticles by inverted fluorescence microscope. The cells were incubated with 20 *μ*M curcumin or Cur-CS nanoparticles, and the cellular uptake was examined at 1, 3, and 6 h time intervals. The electrostatic interaction between nanoparticles and the cell membrane initiates the uptake of nanoparticles into cells, which is strongly affected by the nanoparticle size, degree of deacetylation, and molecular weight and zeta potential [58]. The Cur-CS nanoparticles showed enhanced cellular uptake, where minimal green fluorescence intensity was observed at 1 h, whereas the curcumin-treated cells emitted noticeable fluorescence at 3 h (Figure 3). This may be due to the positive surface charge exhibited by the Cur-CS nanoparticles expressing stronger affinity for the negatively charged cell membrane, enhancing its absorption efficacy in epithelial cells. Furthermore, the interaction of chitosan with the cell membrane results in structural recognition of tight junction-associated proteins, which is followed by enhanced transport through paracellular pathways [59].

### 3.6. Cell Viability

**Figure 4:**
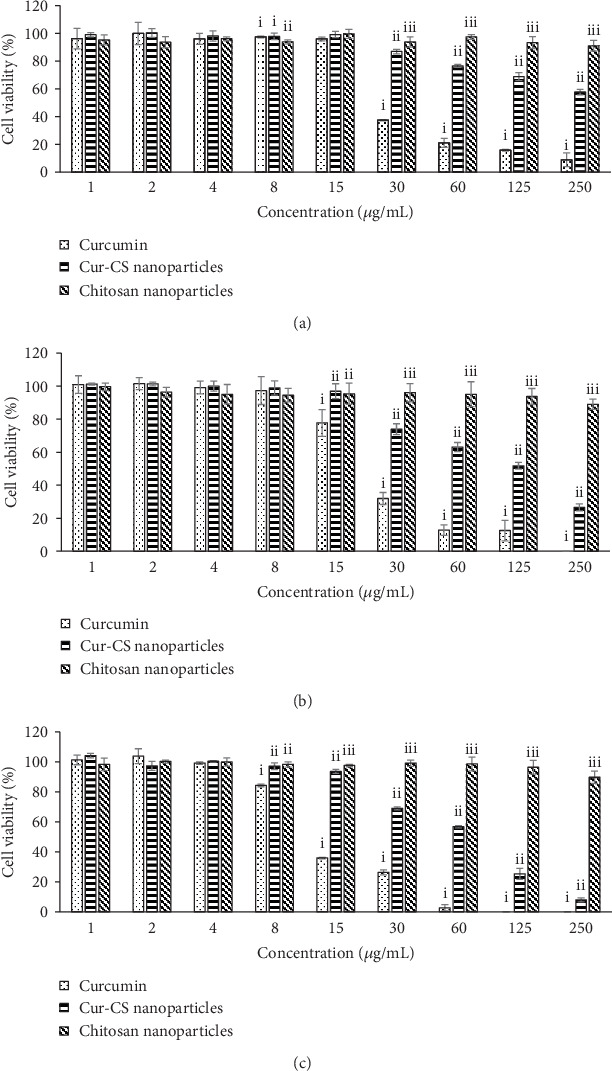
Cell viability (%) for each compound in different concentrations from 1 *μ*g/mL to 250 *μ*g/mL at (a) 24 h, (b) 48 h, and (c) 72 h. For each concentration, means with different roman numerals were significantly different (*p* < 0.05) from each other. CS: chitosan; Cur-CS: curcumin-encapsulated chitosan.

Figure 4 shows that the chitosan nanoparticles caused no cytotoxic effect on CrFK cells for concentrations up to 250 *μ*g/mL. However, there was significantly decreased viability in the cells treated with curcumin and the Cur-CS nanoparticles. The cells tolerated up to 15 *μ*g/mL and 60 *μ*g/mL curcumin and Cur-CS nanoparticles, respectively. The cytotoxic effect of curcumin on different cells has been well demonstrated, where no cytotoxic effect was observed following exposure up to 30 *μ*M curcumin [19, 60, 61]. The MTT results of the present study revealed that the Cur-CS nanoparticles had less cytotoxic effects than curcumin in a dose- and time-dependent manner, suggesting that the chitosan nanoparticles are associated with the reduced cytotoxic effects of curcumin. A previous study has demonstrated that docetaxel-chitosan nanoparticles caused reduced cytotoxicity in MCF-7 cells compared with the free drug, particularly due to the presence of chitosan nanoparticles preventing the drug from acting directly on the cells and the delay in the time required for cleavage of the polymer-drug bond, thus slowing the drug's immediate action [53].

### 3.7. Inhibitory Effect of Curcumin and Cur-CS Nanoparticles on FIPV

The time of addition experiment was used to determine the timing of curcumin in exerting protective effects in virus-infected cells. FIPV-infected CrFK cells were treated at three different stages: before infection (pretreatment), at the same time as virus infection (cotreatment), or at 2 hpi (posttreatment). As tabulated in Table 4, significant protective effects were observed in each stage; however, 20 *μ*M curcumin and Cur-CS nanoparticles showed the highest protection percentage in cotreatment at 48 h, namely, 56% and 68%, respectively. The inhibitory effect was reduced at 72 hpi. Data also indicated enhanced dose-dependent inhibition of viral replication by the Cur-CS nanoparticles as compared to curcumin.  Table 5 lists the CC_50_, EC_50_, and SI for curcumin and Cur-CS nanoparticles. The SI value of the Cur-CS nanoparticles was 11.03, which was threefold higher than that of curcumin (3.72). Curcumin has been studied as an antiviral agent against numerous viruses by interfering with the virus replication cycle [62]. Moreover, curcumin can affect the infectivity of enveloped viruses such as Japanese encephalitis virus and dengue virus type II by disrupting membrane structure integrity during virus-cell attachment [19]. Several studies have demonstrated that nanoformulation of curcumin poses increased bioavailability and enhanced viral inhibitory effects up to threefold against HIV-1 and RSV [30, 62].

### 3.8. Effect of Cur-CS Nanoparticles on Viral Load

Table 6 shows the viral RNA log_10_ copy number and the percentage log_10_ virus copy number decrement compared to virus samples. FIPV replication was inhibited in a concentration-dependent manner, where the highest decrement percentage of the virus copy number was achieved by 20 *μ*M Cur-CS nanoparticles at 48 hpi, which is 73.97 ± 4.45, while 20 *μ*M curcumin significantly decreased the virus copy number to 59.46 ± 2.45 at 48 hpi. Nonetheless, the antiviral effects of both compounds were reduced after 72 hpi. These results suggest that the enhanced inhibitory effects of the Cur-CS nanoparticles on FIPV as compared to curcumin indicate consistency with the results of the MTT antiviral assay. Apart from that, the data showed minimal significant protective effects in the pretreatment and posttreatment assays. One possible explanation is that curcumin is able to interfere with the early stage of virus infection and suppresses viral replication through cellular pathway modulation. Previous studies have described the inhibitory effects of curcumin on several viruses through the regulation of multiple cellular pathways, including the nuclear factor- (NF-) *κ*B signalling pathways and the ubiquitin-proteasome system, and via cellular post-transcriptional and post-translational modifications [63–66].

### 3.9. Effect of Cur-CS Nanoparticles on Proinflammatory Cytokine mRNA Expression

**Figure 5:**
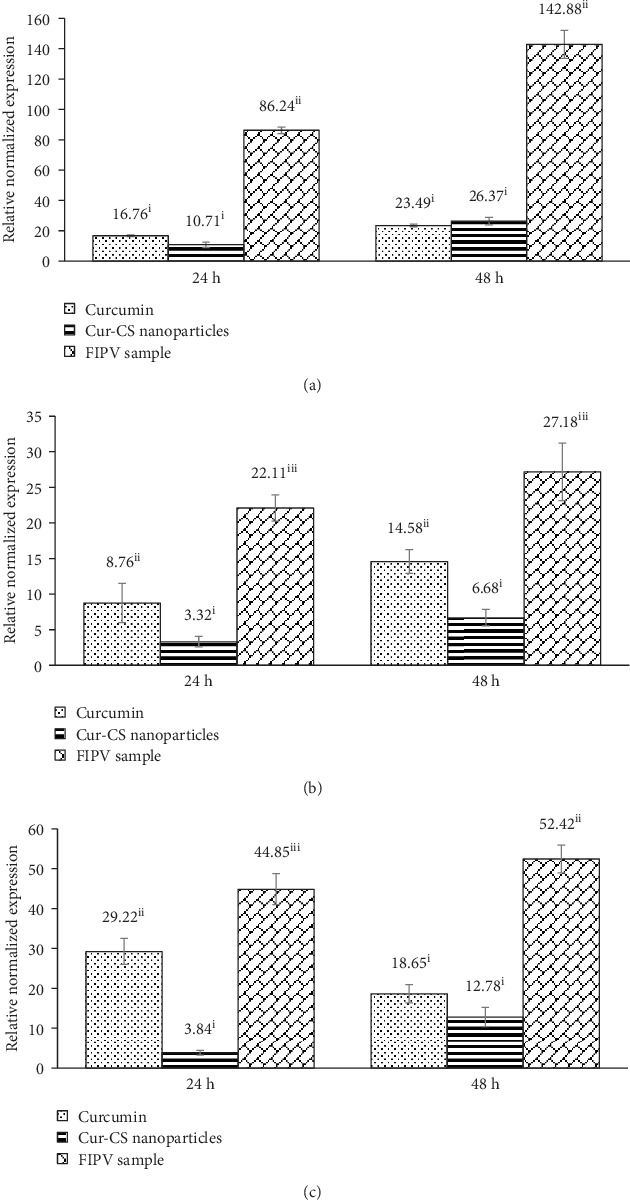
The mRNA expression levels of (a) IL-1*β*, (b) IL-6, and (c) TNF*α* in CrFK cells after FIPV infection with or without treatments at 24 and 48 h. For each compound, means with different roman numerals were significantly different (*p* < 0.05) from each other. Cur-CS: curcumin-encapsulated chitosan; FIPV: feline infectious peritonitis virus.

To examine the influence of curcumin and the Cur-CS nanoparticles on proinflammatory cytokine mRNA expression, IL-1*β*, IL-6, and TNF*α* mRNA levels were evaluated by reverse transcriptase qPCR with GAPDH as the reference gene and cells without virus as the negative control. The IL-1*β*, IL-6, and TNF*α* mRNA expression levels were significantly higher in untreated virus-infected cells; reduced IL-1*β*, IL-6, and TNF*α* mRNA expression levels were noted in cells treated with curcumin and Cur-CS nanoparticles (Figure 5). Curcumin and the Cur-CS nanoparticles exhibited comparable anti-inflammatory effects during FIPV infection. The overwhelming production of inflammatory cytokines is one of the key roles in the pathogenesis of FIP; serositis and pyogranulomatous reaction are the typical immunologic phenomena due to the uncontrolled immune response in infected hosts [5]. The present study demonstrates that curcumin and the Cur-CS nanoparticles significantly inhibited TNF*α*, IL-1*β*, and IL-6 mRNA expression in FIPV-infected cells. The promising anti-inflammatory effects of curcumin have been broadly demonstrated in immune-mediated diseases. The introduction of curcumin to mice infected with human cytomegalovirus reduced TNF*α* and IL-6 production; it also suppressed the oxidative damage caused by the disease [67]. In addition, dietary supplementation of curcumin yields beneficial effects on the inflammatory state in obese cats by lowering the mRNA expression levels of the involved cytokines [68].

### 3.10. Effect of Cur-CS Nanoparticles on Feline Immune-Related Proteins

A feline-specific magnetic bead-based multiplex immunoassay was employed to investigate the effects of curcumin and the Cur-CS nanoparticles against FIPV-infected cells. The concentrations of the immune-related proteins of the treated and untreated samples at 24 and 48 hpi are shown in Tables 7 and 8, respectively. Some proteins were undetectable by the MAGPIX®, as they might not be expressed in epithelial cells; excepting platelet-derived growth factor BB (PDGF-BB), stromal cell-derived factor-1 (SDF-1), monocyte chemoattractant protein-1 (MCP-1), and TNF*α*, the protein concentrations in treated cells at 24 hpi were significantly reduced compared to those in the virus control. Meanwhile, all detectable immune-related proteins in 48 h-treated samples were significantly decreased. The protein concentrations in the virus-inoculated supernatants were increased significantly as compared with those in the cell control at 24 and 48 h, indicating these immune-related proteins were affected during FIPV infection. In agreement with the cytokine mRNA expression results, significant differences were also noted in the concentration of immune-related proteins in cells treated with curcumin and the Cur-CS nanoparticles as compared to the treated FIPV-infected cells. Previous studies have reported that the mechanism underlying the anti-inflammatory activity of curcumin involves the modulation of NF-*κ*B-mediated inflammation and mitogen-activated protein kinase p38 [69, 70].

The present study results reveal that Cur-CS nanoparticles caused less cellular toxicity and exhibited antiviral and anti-inflammatory effects, with an SI value three times higher than that of curcumin. We hypothesised that enhanced cellular uptake promotes the effects of Cur-CS nanoparticles on FIPV-infected cells. Another explanation is that the addition of chitosan nanoparticles can amplify the viral inhibitory and anti-inflammatory effects together with curcumin, as several studies have demonstrated chitosan can improve immunity against viral infections [71, 72]. Nevertheless, further investigations may be needed to scrutinise the actual pathways or mechanisms involved in the curcumin inhibitory effect on FIPV and the factors underlying the enhanced effects of Cur-CS nanoparticles.

### 3.11. Pharmacokinetics Study in Healthy Cats

The employment of chitosan nanoparticles as an efficient delivery system for improving the oral bioavailability of curcumin in cats was investigated using HPLC. No curcumin was detected in cats treated with 50 mg/kg and 100 mg/kg curcumin or Cur-CS nanoparticles at any interval (data not shown). This was mainly due to the curcumin content in the plasma samples being below the detection limit (25 ng/mL) of the HPLC system. Hence, the pharmacokinetics study was performed in cats administered orally with 250 mg/kg curcumin or Cur-CS nanoparticles s.i.d. and b.i.d.

**Figure 6:**
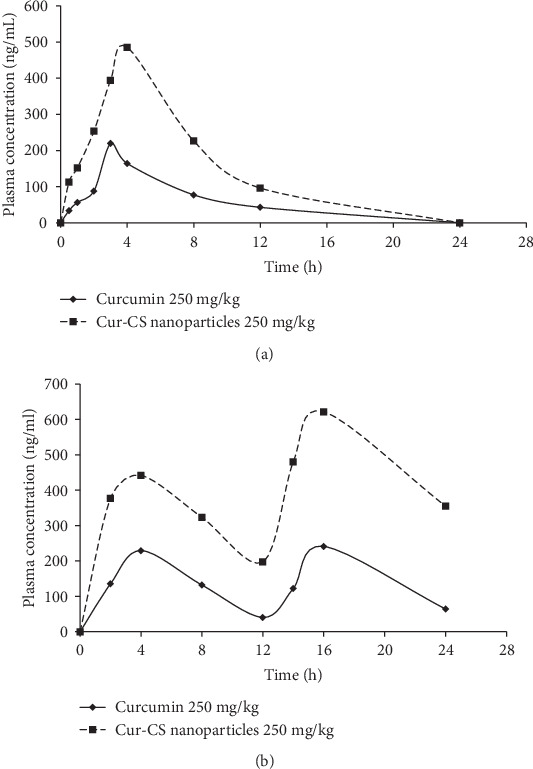
Concentration profile of curcumin in plasma versus time after (a) s.i.d. and (b) b.i.d. oral administration of curcumin or Cur-CS nanoparticles. Cur-CS: curcumin-encapsulated chitosan.

In the s.i.d. pharmacokinetics study, the absorption of orally administered 250 mg/kg curcumin was fairly rapid, where *C*_max_ was detected in the blood at 3 h (Figure 6(a)). Oral administration of 250 mg/kg Cur-CS nanoparticles showed increased absorption, with the *C*_max_ value achieved in 4 h, 1 h later than that for curcumin alone. The curcumin content in the plasma decreased gradually within 24 h, as no curcumin was detected at 24 h postfeeding. A collection of studies in the field have confirmed that employment of chitosan nanoparticles improves the absorption and systemic exposure of poor-bioavailability drugs in plasma [33, 40]. Data suggest that the Cur-CS nanoparticles exhibit higher systemic exposure and promote the oral absorption of curcumin in cats. However, administering Cur-CS nanoparticles s.i.d. did not favour prolonged sustained release of curcumin over 24 h; thus, we conducted the b.i.d. pharmacokinetics study.

In the b.i.d. pharmacokinetics study, multiple peaks were plotted for the mean curcumin plasma concentration versus time profiles (Figure 6(b)). One peak was observed within 0–12 h, and another peak was noted within 12–24 h. The absorption of orally administered 250 mg/kg curcumin and Cur-CS nanoparticles achieved *T*_max_ between 2 and 4 h, which is similar to the s.i.d. pharmacokinetics profile. The *C*_max_ of curcumin treatment was detected at 4 h after the second dose; the Cur-CS nanoparticles achieved an even higher *C*_max_ at the same time point.  Table 9 summarises the relevant pharmacokinetics parameters of the 250 mg/kg curcumin and Cur-CS nanoparticles. The AUC_0-12_ of curcumin and the Cur-CS nanoparticles covered more than 80% of the AUC_0-∞_, indicating these sampling schedules could provide a reliable estimate of the extent of exposure of the treatments. We noted significantly increased *C*_max_, AUC_0-12_, and AUC_0-24_ of the Cur-CS nanoparticles as compared to curcumin either in the s.i.d. or b.i.d. pharmacokinetics study, suggesting that oral delivery with chitosan nanoparticles as a carrier was able to enhance the absorption of curcumin in the blood circulation system in cats. There were no significant differences in the plasma curcumin concentration at both peaks in the curcumin treatment group. Conversely, cats that received 250 mg/kg Cur-CS nanoparticles orally showed a higher *C*_max_ 4 h after administration of the second dose, indicating that twice-daily oral feeding with Cur-CS nanoparticles improves the oral absorption of curcumin more than once-daily oral feeding. The increased curcumin concentration in oral b.i.d. suggests that more than 24 h is needed to achieve a steady state in cats, as a previous study has shown some compounds need 6–7 days of daily dosing to achieve steady-state concentrations in human subjects [73]. However, similar experiments could not be performed, as 250 mg/kg Cur-CS nanoparticles is an excessive amount for cats to take in over 28 consecutive days.

## 4. Conclusion

We examined the effect of Cur-CS nanoparticles in FIPV infection compared to curcumin. Cur-CS nanoparticles exhibited antiviral effects against FIPV *in vitro*. Moreover, this is the first study to investigate the pharmacokinetics of curcumin in cats. The nanoformulation of curcumin with chitosan improved the oral bioavailability of curcumin. Future studies are necessary to investigate the toxicity of Cur-CS nanoparticles in healthy cats and their therapeutic effects as treatment in cats infected with FIPV or other feline diseases.

## Figures and Tables

**Table 1:** Primers sequences for GAPDH, IL-1*β*, IL-6, and TNF*α* mRNA detection.

Gene	Amplicon size (bp)	Sequence (5′ to 3′)	Reference
GAPDH	96	F: AATTCCACGGCACAGTCAAGG	[10]
		R: CATTTGATGTTGGCGGGATC	
IL-1*β*	178	F: CTGGTGCTGTCTGGCTCATA	[10]
		R: TTCCCGTCTTTCATCACACA	
IL-6	333	F: TGCCTGACAAGAATCACTACT	[44]
		R: GAACTACAGCAATCTTAGATG	
TNF*α*	250	F: TGGCCTGCAACTAATCAACC	[10]
		R: GTGTGGAAGGACATCCTTGG	

F: forward; R: reverse.

**Table 2:** Characterisation parameters of chitosan nanoparticles and Cur-CS nanoparticles.

	Chitosan/curcumin ratio	Mean particle size (nm)	Zeta potential (mV)	Encapsulation efficacy (%)	Drug loading (%)
Cur-CS nanoparticles	1 : 1	365.8 ± 15.1^a^	ND	64.3 ± 3.2^a^	31.1 ± 3.9^a^
	1 : 0.75	332.4 ± 9.4^b^	42.1 ± 3.0	77.2 ± 3.6^b^	46.7 ± 2.9^b^
	1 : 0.5	339.4 ± 10.8^b^	ND	81.9 ± 4.5^b^	44.3 ± 2.2^b^
Chitosan nanoparticles		239.2 ± 6.4^c^	53.3 ± 2.6	—	—

Data are presented as mean ± SD (*n* = 3) and analysed using one-way ANOVA and *post hoc* Duncan multiple test. For each parameter, means within a column with different roman letters were significantly different (*p* < 0.05) from each other. Cur-CS: curcumin-encapsulated chitosan; ND: not determined.

**Table 3:** Mean particle size and zeta potential of Cur-CS nanoparticles stored at 4°C and room temperature at definite time intervals.

Day	Mean particle size (nm) at		Zeta potential (mV) at	
	4°C	Room temperature	4°C	Room temperature
0	324.9 ± 9.3	331.1 ± 6.5^a^	44.3 ± 1.8	46.6 ± 1.1
15	331.3 ± 6.0	348.6 ± 8.8^b^	45.0 ± 1.7	44.7 ± 2.5
30	335.3 ± 6.7	359.5 ± 8.9^b^	44.0 ± 2.6	45.4 ± 2.2

Data are presented as mean ± SD (*n* = 3) and analysed using one-way ANOVA and *post hoc* Duncan multiple test. For each temperature, means within a column with different roman letters were significantly different (*p* < 0.05) from each other.

**Table 4:** Percentages of protection in curcumin and Cur-CS nanoparticle treatments with virus in different exposure methods at different concentrations.

Compound	Concentration (*μ*M)	Pretreatment		Cotreatment		Posttreatment	
		48 h	72 h	48 h	72 h	48 h	72 h
Curcumin	2.5	0.00 ± 0.00^a^	0.58 ± 1.00^a^	12.34 ± 2.23^a^	5.76 ± 4.13^a^	0.85 ± 1.35^a^	0.05 ± 0.08^a^
	5	0.38 ± 0.66^a^	1.74 ± 1.06^ab^	21.98 ± 7.01^b^	11.99 ± 3.43^b^	0.08 ± 0.13^a^	0.99 ± 1.71^a^
	10	3.87 ± 1.64^ab^	1.16 ± 1.82^a^	40.08 ± 3.71^d^	18.82 ± 1.93^c^	13.31 ± 3.93^b^	2.59 ± 1.55^a^
	20	12.79 ± 2.27^c^	5.12 ± 2.03^bc^	55.95 ± 1.82^e^	22.25 ± 2.05^c^	17.28 ± 0.78^bc^	2.13 ± 1.91^a^
Cur-CS nanoparticles	2.5	0.98 ± 1.70^a^	0.89 ± 1.30^a^	11.52 ± 2.93^a^	6.29 ± 4.28^a^	0.34 ± 0.59^a^	0.42 ± 0.73^a^
	5	6.53 ± 4.46^b^	1.30 ± 2.26^ab^	32.51 ± 3.53^c^	12.23 ± 3.03^b^	3.87 ± 3.34^a^	1.12 ± 1.01^a^
	10	14.43 ± 6.27^c^	7.98 ± 3.93^c^	49.37 ± 2.95^e^	21.18 ± 1.91^c^	18.02 ± 2.21^c^	3.06 ± 1.00^a^
	20	20.12 ± 3.71^d^	11.67 ± 1.76^d^	67.88 ± 4.08^f^	28.43 ± 3.92^d^	31.25 ± 3.19^d^	12.24 ± 4.46^b^

Data are presented as mean ± SD (*n* = 3) and analysed using one-way ANOVA and *post hoc* Duncan multiple test. For each time interval, means within a column with different roman letters were significantly different (*p* < 0.05) from each other. Cur-CS: curcumin-encapsulated chitosan.

**Table 5:** CC50, EC_50_, and SI values of curcumin and Cur-CS nanoparticles at 48 h.

	CC_50_ (*μ*M)		EC_50_ (*μ*M)		SI	
	Curcumin	Cur-CS nanoparticles	Curcumin	Cur-CS nanoparticles	Curcumin	Cur-CS nanoparticles
24 h	69.9	237.5	—	—	—	—
48 h	59.1	111.4	15.9	10.1	3.72	11.03
72 h	35.5	70.5	—	—	—	—

Cur-CS: curcumin-encapsulated chitosan; SI: selective index.

**Table 6:** Log_10_ copy number of virus and decrement percentages for 3'UTR log_10_ copy number in samples treated for 24, 48 and 72 h.

Incubation time	Treatment	3′UTR log_10_ copy number	% log_10_ copy number decrement
48 h	FIPV sample	11.86 ± 0.06^e^	0.00 ± 0.00^a^
	Curcumin 5 *μ*M	11.72 ± 0.06^d^	27.54 ± 3.28^b^
	Cur-CS 5 *μ*M	11.65 ± 0.05^d^	37.30 ± 1.14^c^
	Curcumin 10 *μ*M	11.63 ± 0.01^cd^	40.50 ± 5.41^c^
	Cur-CS 10 *μ*M	11.54 ± 0.02^bc^	51.38 ± 5.50^d^
	Curcumin 20 *μ*M	11.46 ± 0.05^b^	59.46 ± 2.45^e^
	Cur-CS 20 *μ*M	11.27 ± 0.11^a^	73.97 ± 4.45^f^
72 h	FIPV sample	12.00 ± 0.03^d^	0.00 ± 0.00^a^
	Curcumin 5 *μ*M	11.95 ± 0.01^cd^	10.70 ± 5.47^b^
	Cur-CS 5 *μ*M	11.94 ± 0.02^bcd^	11.72 ± 5.53^b^
	Curcumin 10 *μ*M	11.86 ± 0.06^abc^	26.92 ± 5.93^cd^
	Cur-CS 10 *μ*M	11.85 ± 0.08^ab^	28.29 ± 7.73^cd^
	Curcumin 20 *μ*M	11.87 ± 0.04^abc^	26.38 ± 2.75^c^
	Cur-CS 20 *μ*M	11.80 ± 0.06^a^	36.33 ± 4.66^d^

Data are presented as mean ± SD (*n* = 3) and analysed using one-way ANOVA and *post hoc* Duncan multiple test. For each time interval and parameter, means within a column of determined incubation time (48 and 72 h) with different roman letters were significantly different (*p* < 0.05) from each other. Cur-CS: curcumin-encapsulated chitosan; FIPV: feline infectious peritonitis virus.

**Table 7:** Measurement of immune-related protein concentrations (pg/mL) of different treatment groups in infected cells at 24 h.

Cytokine/chemokine	Uninfected control cells	Infected cells	Infected cells treated with curcumin	Infected cells treated with Cur-CS nanoparticles
PDGF-BB	350.4 ± 7.7^a^	445.6 ± 18.7^b^	446.0 ± 0.0^b^	439.3 ± 0.0^b^
IL-8	222.4 ± 18.7^a^	2078.0 ± 138.6^d^	1509.5 ± 16.3^c^	1153.0 ± 2.8^b^
KC	17.26 ± 12.84^a^	80.76 ± 3.71^c^	58.52 ± 15.92^bc^	46.15 ± 1.58^b^
SDF-1	76.3 ± 0.0^a^	161.3 ± 7.1^b^	163.5 ± 3.1^b^	155.3 ± 4.1^b^
RANTES	15.9 ± 6.5^a^	868.6 ± 62.1^d^	746.7 ± 18.3^c^	647.6 ± 29.9^b^
MCP-1	735.5 ± 34.2^a^	934.8 ± 0.0^c^	934.8 ± 0.0^c^	893.3 ± 0.0^bc^
TNF*α*	26.97 ± 1.07^a^	38.13 ± 0.00^b^	37.40 ± 1.04^b^	35.18 ± 2.09^b^
IL-18	<31.53	60.62 ± 3.50^c^	47.92 ± 1.73^ab^	42.68 ± 1.13^a^

Data are presented as mean ± SD where each experiment was performed in duplicate and analysed using one-way ANOVA and *post hoc* Duncan multiple test. For each cytokine/chemokine, means within a row with different roman letters were significantly different (*p* < 0.05) from each other. Cur-CS: curcumin-encapsulated chitosan.

**Table 8:** Measurement of immune-related protein concentrations (pg/mL) of different treatment groups in infected cells at 48 h.

Cytokine/chemokine	Uninfected control cells	Infected cells	Infected cells treated with curcumin	Infected cells treated with Cur-CS nanoparticles
PDGF-BB	326.8 ± 8.81^a^	440.5 ± 43.1^b^	354.2 ± 30.0^a^	350.0 ± 7.7^a^
IL-6	<37.42	108.06 ± 4.85	54.75 ± 6.34^∗^	<39.42
IL-8	423.3 ± 18.5^a^	1660.0 ± 196.6^c^	1074.5 ± 9.2^b^	641.0 ± 28.4^a^
KC	21.0 ± 7.5^a^	266.1 ± 35.2^d^	186.4 ± 14.2^c^	68.0 ± 0.0^b^
SDF-1	69.1 ± 10.2^a^	158.2 ± 0.9^b^	84.2 ± 2.9^a^	69.9 ± 11.4^a^
RANTES	24.2 ± 5.3^a^	2317.0 ± 355.0^c^	618.6 ± 70.8^b^	501.1 ± 69.1^b^
MCP-1	711.4 ± 0.0^a^	934.2 ± 57.9^b^	735.5 ± 34.2^a^	686.0 ± 35.8^a^
TNF*α*	27.72 ± 0.00^a^	46.83 ± 6.11^b^	35.18 ± 2.09^a^	26.97 ± 1.07^a^

Data are presented as mean ± SD where each experiment was performed in duplicate and analysed using one-way ANOVA and *post hoc* Duncan multiple test. For each cytokine/chemokine, means within a row with different roman letters were significantly different (*p* < 0.05) from each other. Mean with an asterisk (∗) was significantly (*p* < 0.05) different from the positive control (infected cells). Cur-CS: curcumin-encapsulated chitosan.

**Table 9:** Pharmacokinetic parameters of curcumin and Cur-CS nanoparticles given s.i.d. and b.i.d. orally in healthy cats.

Formulation	Curcumin	Cur-CS nanoparticles
*Single dosing (s.i.d.)*		
*C*_max_ (ng/mL)	219.6 ± 18.6	485.0 ± 36.2^∗^
*T*_max_ (h)	3	4
AUC_0-12_ (ng/mL h)	1139.3 ± 136.7	3015.6 ± 385.7^∗^
AUC_0-∞_ (ng/mL h)	1374.5 ± 112.1	3476.0 ± 505.3^∗^
*Two dosing (b.i.d.)*		
*C*_max_ (ng/mL)	241.1 ± 27.1	621.5 ± 38.2^∗^
*T*_max_ (h)	4	4
AUC_0-12_ (ng/mL h)	1508.0 ± 72.4	3733.1 ± 132.9^∗^
AUC_0-24_ (ng/mL h)	3098.9 ± 176.2	9316.9 ± 169.0^∗^

Data are presented as mean ± SD (*n* = 4) and analysed using paired sample *t*-test. For each parameter, means with an asterisk (^∗^) were significantly (*p* < 0.05) different from the control (curcumin). Cur-CS: curcumin-encapsulated chitosan.

## Data Availability

The datasets used and/or analyzed during the current study are available from the corresponding author on reasonable request.

## Abbreviations

AUC: Area under the curve
b.i.d.: Two dosing
CC_50_: Median cytotoxic concentration
CrFK: Crandell-Rees feline kidney
cDNA: Complementary DNA
*C*_max_: Maximum plasma concentration
CPE: Cytopathic effect
Cur-CS: Curcumin-encapsulated chitosan
DMSO: Dimethyl sulfoxide
EC_50_: Median effective concentration
FCoV: Feline coronavirus
FeLV: Feline leukaemia virus
FIP: Feline infectious peritonitis
FIPV: FIP virus
FIV: Feline immunodeficiency virus
GAPDH: Glyceraldehyde-3-phosphate dehydrogenase
h: Hours
HIV: Human immunodeficiency virus
hpi: Hours postinfection
HPLC: High-performance liquid chromatography
IL: Interleukin
MEM: Minimum essential medium
min: Minutes
MTT: 3-(4,5-Dimethyl-2-thiazolyl)-2,5-diphenyl-2H-tetrazolium bromide
NaOH: Sodium hydroxide
NEAA: Nonessential amino acid
OD: Optical density
PBS: Phosphate buffered saline
s: Seconds
SARS-CoV: Severe acute respiratory syndrome coronavirus
SD: Standard deviation
SI: Selective index
s.i.d.: Single dosing
TCID_50_: Tissue culture infective dose 50
TEM: Transmission electron microscope
*T*_max_: Time to reach maximum plasma concentration
TNF: Tumour necrosis factor
TPP: Tripolyphosphate
UV-Vis: Ultraviolet-visible.
